# Over-Expression of Catalase in Myeloid Cells Confers Acute Protection Following Myocardial Infarction

**DOI:** 10.3390/ijms15059036

**Published:** 2014-05-21

**Authors:** E. Bernadette Cabigas, Inthirai Somasuntharam, Milton E. Brown, Pao Lin Che, Karl D. Pendergrass, Bryce Chiang, W. Robert Taylor, Michael E. Davis

**Affiliations:** 1 Wallace H. Coulter Department of Biomedical Engineering, Emory University and Georgia Institute of Technology, Atlanta, GA 30322, USA; E-Mails: bgcab@yahoo.com (E.B.C.); inthirai3@gatech.edu (I.S.); mebrow5@emory.edu (M.E.B.); paolin.che@gmail.com (P.L.C.); kpdb35@hotmail.com (K.D.P.); bryce.chiang@emory.edu (B.C.); w.robert.taylor@emory.edu (W.R.T.); 2 Division of Cardiology, Emory University School of Medicine, Atlanta, GA 30322, USA; 3 Emory Children’s Center for Cardiovascular Biology, Children’s Healthcare of Atlanta, Atlanta, GA 30322, USA

**Keywords:** oxidative stress, myocardial infarction, antioxidant therapy

## Abstract

Cardiovascular disease is the leading cause of death in the United States and new treatment options are greatly needed. Oxidative stress is increased following myocardial infarction and levels of antioxidants decrease, causing imbalance that leads to dysfunction. Therapy involving catalase, the endogenous scavenger of hydrogen peroxide (H_2_O_2_), has been met with mixed results. When over-expressed in cardiomyocytes from birth, catalase improves function following injury. When expressed in the same cells in an inducible manner, catalase showed a time-dependent response with no acute benefit, but a chronic benefit due to altered remodeling. In myeloid cells, catalase over-expression reduced angiogenesis during hindlimb ischemia and prevented monocyte migration. In the present study, due to the large inflammatory response following infarction, we examined myeloid-specific catalase over-expression on post-infarct healing. We found a significant increase in catalase levels following infarction that led to a decrease in H_2_O_2_ levels, leading to improved acute function. This increase in function could be attributed to reduced infarct size and improved angiogenesis. Despite these initial improvements, there was no improvement in chronic function, likely due to increased fibrosis. These data combined with what has been previously shown underscore the need for temporal, cell-specific catalase delivery as a potential therapeutic option.

## 1. Introduction

Myocardial infarction affects millions of patients annually and leads to heart failure, for which there is no cure [1]. Following ischemic injury in the heart, there is an overproduction of reactive oxygen species (ROS) that leads to tissue damage, fibrosis, prevents angiogenesis, and limits regeneration [2–5]. Of the ROS, the two major species implicated in these processes are superoxide and hydrogen peroxide (H_2_O_2_). Both are increased following infarction and levels of their endogenous scavengers progressively decrease [6,7]. Catalase catalyzes the conversion of H_2_O_2_ to water and oxygen and therefore renders it less toxic to cells. It is one of the most important peroxidases in the myocardium, potentially making up 80% of all peroxidase activity in cardiomyocytes [8]. Following ischemic injury in the myocardium, there are mixed results of the therapeutic potential of catalase. Delivery of catalase was shown to protect both by itself, and when used in combination with superoxide dismutase [9–11]. In other studies catalase was shown to have no benefit, thus there is much confusion on the effects of catalase as a potential therapeutic intervention [12–14].

Adding to this controversy is the finding that, in small amounts, H_2_O_2_ acts to initiate a cascade of protective pathways that is activated during oxidative stress [15]. In many cell types, H_2_O_2_ is required for normal function and excessive amounts can even promote cardiogenic differentiation of stem cells [16,17]. When catalase was over-expressed in mouse hearts from birth, there was a significant improvement in cardiac function following infarction as compared to their wild-type littermates [18,19]. When catalase was over-expressed in cardiomyocytes in an inducible manner, no acute effect was seen, though cardiac function improved chronically due to altered remodeling, indicating the need for temporal management [20]. Recent data demonstrated that when H_2_O_2_ is absent in myeloid cells (due to targeted over-expression of catalase), macrophage recruitment and infiltration, as well as angiogenesis were impaired in a model of hindlimb ischemia [21]. Thus it would appear that the role of catalase is both time- and cell-specific and these could all lead to conflicts in therapeutic efficacy depending on protein *versus* gene delivery, and localization effects.

Given previous opposing effects of cardiomyocyte-specific catalase over-expression, and the potential role of inflammatory cell-derived H_2_O_2_ in the ischemic healing process, this study sought to determine the role of myeloid-specific over-expression of catalase on post-infarct healing. Mice over-expressing human catalase under control of a myeloid-specific promoter were subjected to myocardial infarction and both acute and chronic healing were determined. Potential mechanisms demonstrated a role for both alterations in angiogenesis, and fibrosis, and also revealed cell-specific responses in the context of prior studies.

## 2. Results and Discussion

### 2.1. Hydrogen Peroxide Production Reduced in Tg^(MLC−CAT)^ Mice Following Myocardial Infarction (MI)

**Figure 1.**
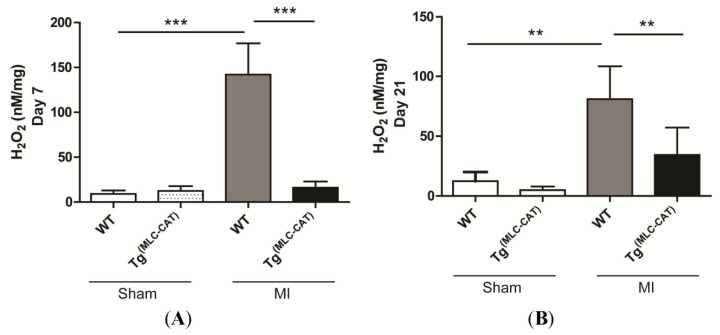
Left ventricular H_2_O_2_ production significantly decreased in Tg^(MLC−CAT)^ mice as compared to wild-type (WT) mice. Measurement of H_2_O_2_ production using Amplex red assay demonstrated a significant increase in H_2_O_2_ levels at both (**A**) 7 and (**B**) 21 days in WT mice *vs.* sham controls. Tg^(MLC−CAT)^ mice had significantly less production of H_2_O_2_. Values are mean ± SEM; *n* = 5–6 per group ** *p* < 0.01 and *** *p* < 0.001 respectively; Analysis of variance (ANOVA) followed by Bonferroni post-test.

To determine if levels of cardiac hydrogen peroxide (H_2_O_2_) production following MI differed in Tg^(MLC−CAT)^ mice, Amplex Red assay was performed using left ventricular tissue and normalized to total tissue weight. H_2_O_2_ levels were significantly elevated at both 7- (*p* = 0.001) and 21-days (*p* < 0.0001) post-infarction in wild-type (WT) mice compared with sham operated controls. Tg^(MLC−CAT)^ mice demonstrated a significant decrease in H_2_O_2_ production compared to their WT littermates at both 7 and 21 days (142.0 ± 34.8 *vs.* 16.1 ± 6.7 nM/mg and 139.4 ± 25.3 *vs.* 40.4 ± 10.6 nM/mg, respectively; *p* < 0.01; Figure 1).

### 2.2. Catalase and Peroxidase Activity Significantly Increased in Tg^(MLC−CAT)^ Mice

**Figure 2.**
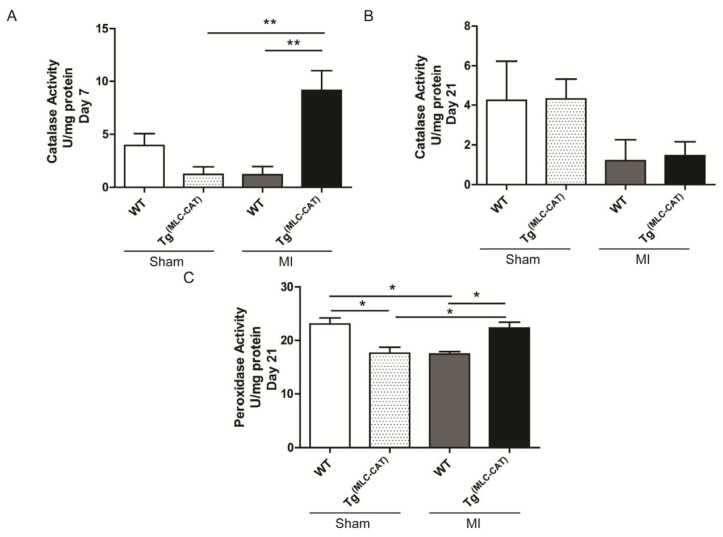
Catalase activity acutely increased in Tg^(MLC−CAT)^ mice as compared to WT mice. (**A**) Catalase activity was significantly increased at 7 days following myocardial infarction in Tg^(MLC−CAT)^ mice as compared to WT mice. This increase in activity was also significant as compared to sham operated animals. Values are mean ± SEM; *n* = 5 per group; ** *p* < 0.01 ANOVA followed by Bonferroni post-test; (**B**) At 21 days, catalase activity in Tg^(MLC−CAT)^ mice showed no significant difference from WT mice activity levels. Values are mean ± SEM; *n* = 5–7 per group; (**C**) Non-catalase peroxidase activity was significantly increased in Tg^(MLC−CAT)^ mice as compared to WT mice at 21 days. Values are mean ± SEM; *n* = 5–8 per group; * *p* < 0.05 ANOVA followed by Bonferroni post-test.

To determine if catalase activity corresponded with the diminished levels of H_2_O_2_ production observed in Tg^(MLC−CAT)^ mice, decomposition of H_2_O_2_ was measured in left ventricular protein homogenates. Catalase activity was increased at 7 days post-infarction (*p* = 0.001) in Tg^(MLC−CAT)^ mice as compared to WT littermates (9.1 ± 1.9 *vs.* 1.2 ± 0.8 U/mg; *p* < 0.05; Figure 2A,B). At 21 days post-infarction, there were no significant changes in any group, though mice subjected to MI had a decreased trend. To determine if other peroxidase activity was altered, decomposition of H_2_O_2_ that was not inhibited by 3-AT (a catalase inhibitor) was determined. There was a significant increase (*p* = 0.002) in non-catalase peroxidase activity in Tg^(MLC−CAT)^ mice subjected to MI as compared to WT mice (22.3 ± 1.1 *vs.* 17.4 ± 0.5 U/mg; *p* < 0.05; Figure 2C).

### 2.3. Catalase Increased in CD45^+^ Cells in Tg^(MLC−CAT)^ Mice Following Infarction

**Figure 3.**
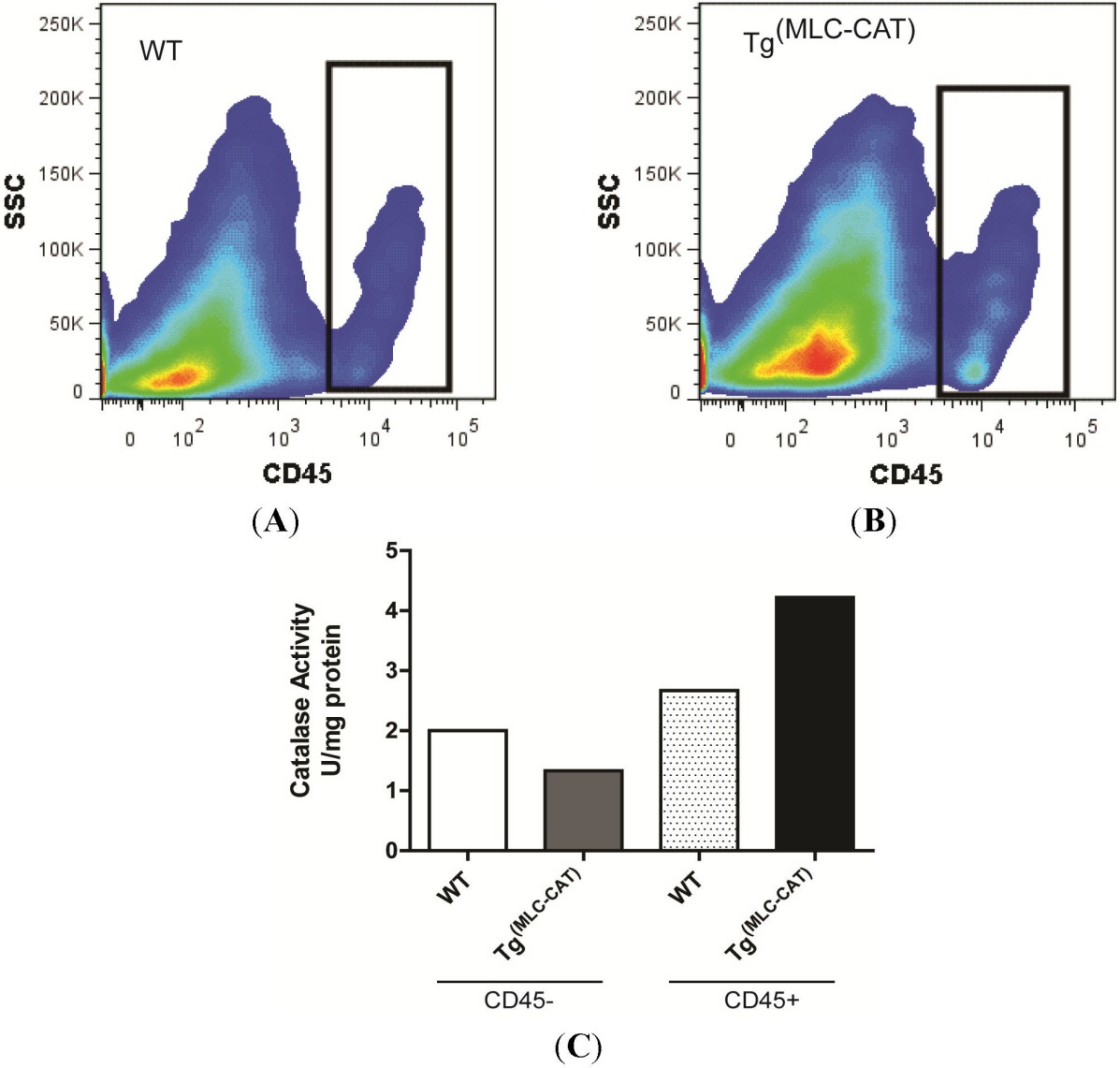
Catalase activity increased in CD45^+^ cells in Tg^(MLC−CAT)^ mice. Cells were isolated from the left-ventricle 7 days following infarction and collected for analysis of catalase activity. (**A**,**B**) Representative cell sorting images showing the fraction of CD45^+^ cells that were collected did not differ between groups (rectangle area denotes CD45^+^ gate); (**C**) Catalase activity in isolated cells demonstrated a trend for increased activity only in CD45^+^ cells of Tg^(MLC−CAT)^ mice while CD45^−^ cells showed no increase. Data are expressed as U/mg protein from pooled samples (*n* = 3 per group).

To determine the cell type responsible for the difference in catalase activity, CD45^+^ cells were isolated from the left-ventricular tissue 7 days following infarction. Figure 3A,B shows a representative cell sorting image used to collect CD45^+^ cells. Catalase activity was measured in both CD45^+^ and CD45^−^ fractions in WT and Tg^(MLC−CAT)^ mice. While there was no change in catalase activity in WT mice, there was a 3-fold increase in the CD45^+^ fraction (Figure 3C) in Tg^(MLC−CAT)^ mice compared to the CD45^−^ fraction. The CD45^+^ fraction of Tg^(MLC−CAT)^ mice also had higher activity than that of WT mice.

### 2.4. Acute Function Is Improved in Tg^(MLC−CAT)^ Mice Following Infarction

**Figure 4.**
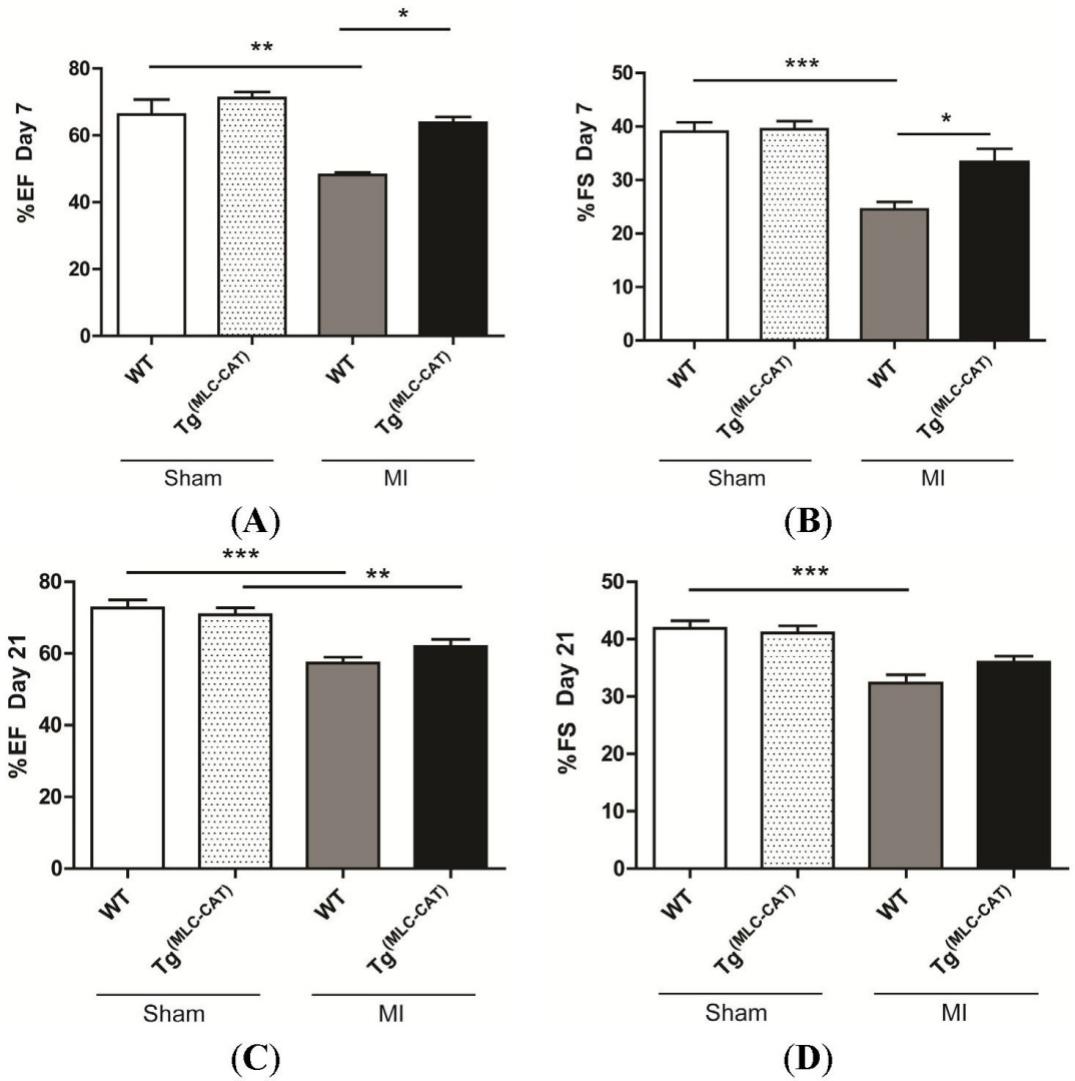
Ventricular function acutely improved in Tg^(MLC−CAT)^ mice. Echocardiographic studies demonstrated: (**A**) At 7 days following myocardial infarction, percent ejection fraction (% ejection fraction (EF)) was significantly increased in Tg^(MLC−CAT)^ mice as compared to WT mice. Values are mean ± SEM; *n* ≥ 6 per group;* *p* < 0.05, ** *p* < 0.01 ANOVA followed by Bonferroni post-test; (**B**) At 7 days following myocardial infarction, percent fractional shortening (% fractional shortening (FS%)) was significantly increased in Tg^(MLC−CAT)^ mice as compared to WT mice. Values are mean ± SEM; *n* ≥ 6 per group; * *p* < 0.05, *** *p* < 0.001 ANOVA followed by Bonferroni post-test; (**C**) At 21 days following myocardial infarction, %EF showed no significant difference between Tg^(MLC−CAT)^ mice as compared to WT mice. Values are mean ± SEM; *n* = 6–9 per group; ** *p* < 0.01, *** *p* < 0.001 ANOVA followed by Bonferroni post-test; (**D**) At 21 days following myocardial infarction, FS% showed no significant difference between Tg^(MLC−CAT)^ mice as compared to WT mice. Values are mean ± SEM; *n* = 7–10 per group; *** *p* < 0.001 ANOVA followed by Bonferroni post-test.

To determine differences in acute ventricular function following permanent ligation, echocardiography was performed 7 days following injury. There was a significant improvement in ejection fraction (EF; *p* = 0.0002) and fractional shortening (FS; *p* = 0.0002) in Tg^(MLC−CAT)^ mice compared to their WT littermates at 7 days post-infarction (EF 63.7% ± 1.8% *vs.* 48.2% ± 0.8%; *p* < 0.05; FS 33.2% ± 2.6% *vs.* 24.5% ± 1.4%; *p* < 0.05). Additionally, WT mice demonstrated significant decreases in function with infarction (*p* < 0.001) in both indices, while Tg^(MLC−CAT)^ mice had no significant decrease with injury (Figure 4A,B). To determine chronic changes in function, echocardiography was performed at 21 days following injury. There were no significant differences between WT and Tg^(MLC−CAT)^ mice in either EF or FS at this time point (Figure 4C,D).

### 2.5. Infarct Size Significantly Decreased in Tg^(MLC−CAT)^ Mice

**Figure 5.**
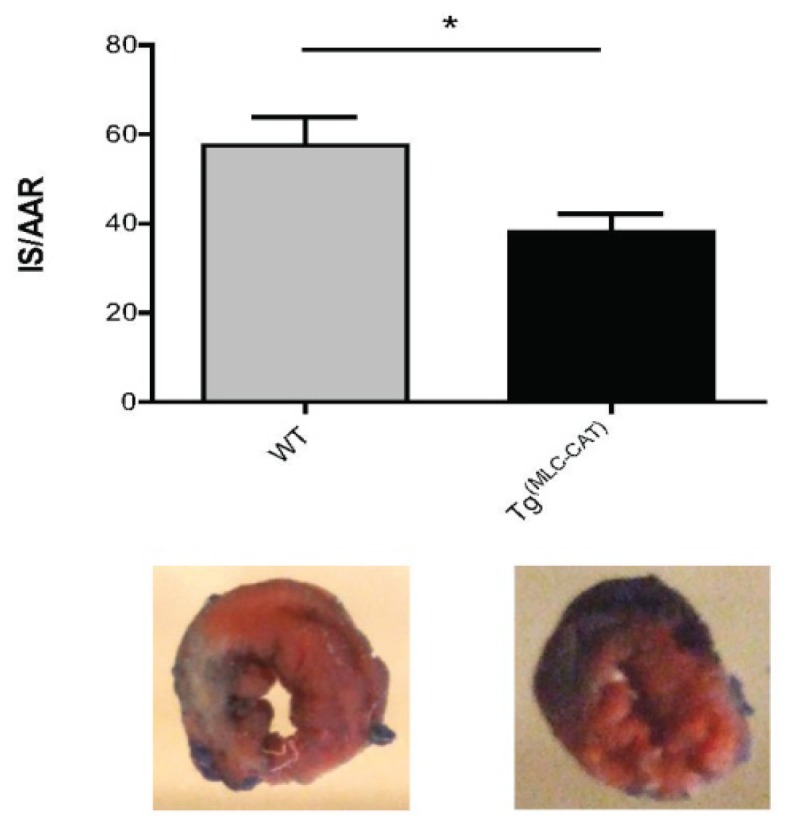
Decrease in infarct size in Tg^(MLC−CAT)^ mice. At day 3 following myocardial infarction, a significant decrease in infarct size was observed in Tg^(MLC−CAT)^ mice as compared to WT mice. Values are mean ± SEM expressed as infarct size/area at risk; *n* = 5–6 per group; * *p* < 0.05 *t*-test.

To determine the degree of tissue damage, infarct size was compared between Tg^(MLC−CAT)^ mice and their WT littermates 72 h following injury. While there were no changes in area-at-risk, there was a significant decrease in infarct size in Tg^(MLC−CAT)^ mice *vs.* WT controls (38.1% ± 4.0% *vs.* 57.5% ± 6.4%; *p* < 0.05; Figure 5).

### 2.6. Increase in Endothelial Cell Infiltration and Vessel Formation in Tg^(MLC−CAT)^ Mice

**Figure 6.**
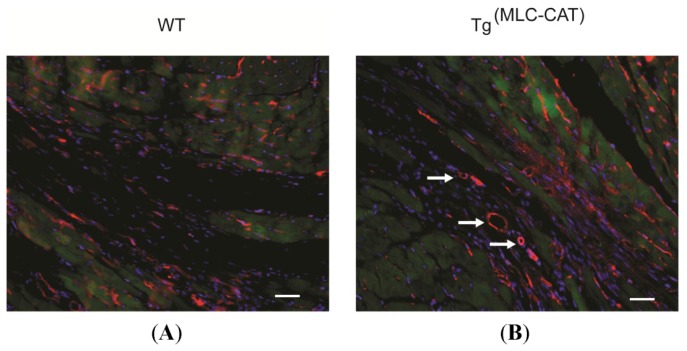

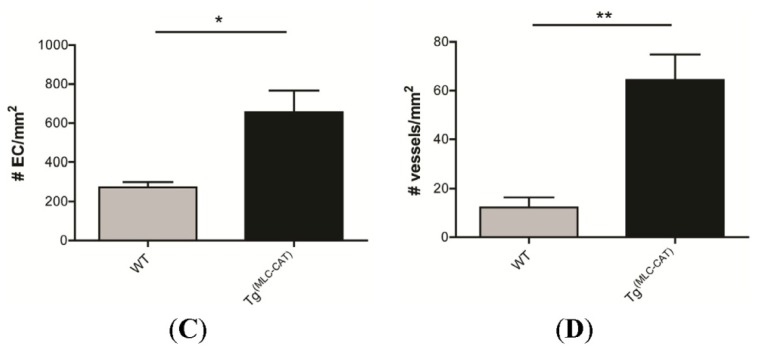
Increase in endothelial cells and vessels in Tg^(MLC−CAT)^ mice. (**A**,**B**) Representative images of infarct tissue stained with isolectin for endothelial cells (red = isolectin; green = autofluorescence; blue = DAPI). White arrows point to vessels, scale bars = 50 μm; (**C**) Quantitative assessment of numbers of vessels and (**D**) endothelial cells, were increased in Tg^(MLC−CAT)^ mice as compared to WT mice at 21 days following myocardial infarction. Values are mean ± SEM; *n* = 4 per group; * *p* < 0.05, ** *p* < 0.01 *t*-test.

To determine the contribution of angiogenesis following myocardial infarction (MI), endothelial cell presence and vessel formation were measured tissue sections following staining with fluorescent Isolectin B4. Data were expressed as either vessels or cells per mm^2^ after counting several sections per animal (representative sections in Figure 6A,B). Vessel formation was significantly increased in Tg^(MLC−CAT)^ mice as compared to WT controls, 64.3 ± 10.5 *vs.* 12.3 ± 4.1, *p* < 0.05; Figure 6C). Endothelial cell number was also increased significantly (655.4 ± 111.6 *vs.* 272.7 ± 26.5; *p* < 0.01; Figure 6D).

### 2.7. Increase in Fibrosis in Tg^(MLC−CAT)^ Mice

**Figure 7.**
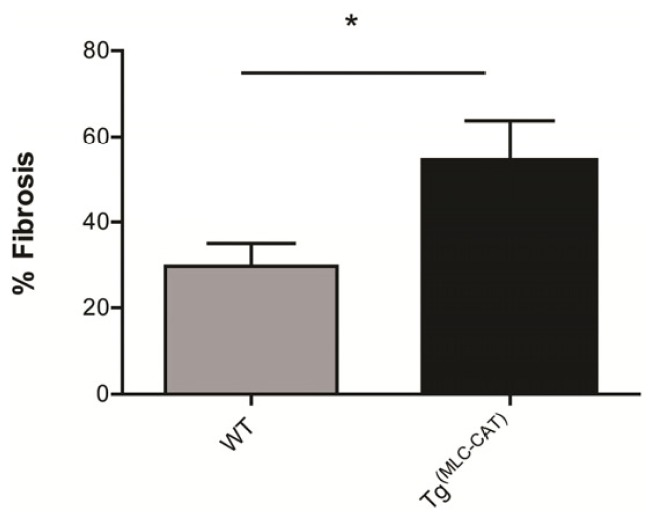
Increase in fibrosis Tg^(MLC−CAT)^ mice. Quantitative assessment of fibrosis (expressed as percent of left ventricle) was increased in Tg^(MLC−CAT)^ mice as compared to WT mice. Values are mean ± SEM; *n* = 4–5 per group; * *p* < 0.05 *t*-test.

To determine the amount of scar tissue formation following MI, fibrosis (collagen formation) was measured by picrosirius red staining and expressed as percent of left ventricle (LV) area. There was very little fibrosis in sham mice (data not shown), however, Tg^(MLC−CAT)^ mice demonstrated an increased level of staining as compared to WT littermates (54.3 ± 9.5 *vs.* 29.7 ± 5.3; *p* < 0.05; Figure 7).

### 2.8. Potential Cytokine/Chemokine Mediators of Changes in Tg^(MLC−CAT)^ Mice

To determine potential signaling molecules that could contribute to the phenotype seen in Tg^(MLC−CAT)^ mice, we collected protein samples after day 7 and ran an enzyme-linked immunosorbent assay (ELISA) array for mediators of inflammation. Samples from 5 mice per group were pooled and data are expressed as OD450/μg protein. As the data in Table 1 shows, there was an increase in pro-inflammatory cytokine levels in WT mice subjected to MI (TNFα, IL-1β, IL-2, MCP1, RANTES). Increases in these cytokines were blunted in Tg^(MLC−CAT)^ mice. Moreover, Tg^(MLC−CAT)^ mice showed increases in pro-angiogenic and pro-survival growth factors such as VEGF, IGF-1, and FGF. Levels of stem cell mobilizing factors were also increased in Tg^(MLC−CAT)^ mice (G-CSF, SCF). Finally, some molecules were either unchanged by MI, or not blunted in Tg^(MLC−CAT)^ mice including IFNγ, IL-17a, and IL-1α.

### 2.9. Discussion

There are mixed studies on the efficacy of delivered catalase following myocardial infarction. While some studies suggest an acute benefit, others show no improvements in function [9–14,22]. Adding to this controversy, studies of over-expression of catalase show differences depending on the timing of over-expression. When expressed by cardiomyocytes from birth, catalase confers both acute and chronic protection [19]. When an inducible system was used to control timing of expression in cardiomyocytes, different results were obtained that demonstrated the need for temporal control [20]. While both of those studies were done with cardiomyocyte-specific over-expression, no studies have been done to determine the role of myeloid-specific over-expression of catalase on post-infarct healing. The goal of this study was to determine whether myeloid-specific over-expression of catalase improves function following infarction.

MI triggers an inflammatory response which results in activation of cytokine and chemokine cascades, leading to recruitment of the primary responders of myeloid lineage (neutrophils and monocytes/macrophages) to the infarct zone within a day of ischemic injury. These cells carry out phagocytosis and wound debridement to clear dead cells and matrix debris. Pro-inflammatory cytokine/chemokine cascades and excessive production of reactive oxygen species (ROS) are key characteristics of this phase [23–25]. While it is acknowledged that ROS may be required for normal tissue balance and healing, excessive production triggers apoptosis/necrosis, impairs tissue regeneration, and leads to adverse remodeling [3,5,26,27]. It was not surprising therefore, that the current study demonstrated acute reduction in H_2_O_2_ levels that correlated with increased levels of catalase. Inflammatory cells responding to the injury were likely present in the heart and seemingly were the main contributors to this finding as shown by catalase activity in CD45^+^ cells from the infarcted LV. Interestingly, while H_2_O_2_ levels were still decreased at 21 days following injury, catalase levels had fallen back to WT levels. At this 3 week time point, these macrophages and myeloid-lineage cells were likely cleared from the site of injury as we could not detect any by flow cytometry. As H_2_O_2_ may also regulate expression of catalase, it is possible that the excessive scavenging of H_2_O_2_ led to a refractory decrease in catalase expression and activity [28,29]. Interestingly, while catalase activity was reduced, there was still significant scavenging of H_2_O_2_ in myocardial tissue. Measurement of non-catalase peroxidase activity was increased, suggesting possible compensatory upregulation of other H_2_O_2_ scavenging enzymes, such as glutathione peroxidases or similar antioxidants.

To determine the effect of this cell-specific scavenging of H_2_O_2_ levels on cardiac function, we performed echocardiography at both 7 and 21 days, representing acute and chronic time-points, respectively. Both measurements of ejection fraction and fractional shortening demonstrated significant improvements in LV function at the 7 day time point in Tg^(MLC−CAT)^ mice as compared to WT littermates. This is in contrast to our prior studies with catalase over-expression in cardiomyocytes where there was no acute improvement in cardiac function [20]. Tg^(MLC−CAT)^ mice also had reduced infarct size, indicating a potential mechanism of cardiomyocyte survival at this early time point. It is quite interesting that over-expression of catalase in cardiomyocytes did not protect the myocytes from acute injury, whereas catalase over-expression in myeloid cells did. Whether this is due to reduced H_2_O_2_ directly on cardiomyocytes or reduced negative paracrine factor release as a result of catalase over-expression is not known, and a potential area for future investigation. Indeed, we saw decreased inflammatory cytokine levels in Tg^(MLC−CAT)^ mice including TNFα, IL-1β, IL-17, MCP-1, and Rantes. Despite initial improvements in cardiac function, and despite continued scavenging of H_2_O_2_, there was no improvement in chronic cardiac function in Tg^(MLC−CAT)^ mice. Once again, this was in stark contrast to our prior study with cardiomyocyte-specific over-expression of catalase where chronic function was improved due to alterations in fibrosis and collagen expression. In the current study, reduction of H_2_O_2_ levels did not lead to a decrease in fibrosis, but rather an increase. It is evident that scavenging of H_2_O_2_ of catalase may not be the primary factor, but rather the cell type in which it is scavenged. While overall H_2_O_2_ levels were decreased in the current study, it is possible that cardiomyocyte H_2_O_2_ was normal or even increased, leading to increased collagen deposition. A previous swine study demonstrated similar increased fibrosis in spite of diminished infarct size and improved ejection fraction with peptide delivery following induction of myocardial infarction [30]. It appears that although ventricular function was acutely improved by treatment with a recombinant peptide, the increased fibrosis carried long-term damaging sequelae. One interesting finding in our study was the discovery that Leptin was highly expressed in Tg^(MLC−CAT)^ mice, almost 10x greater than WT levels. A recent study found that Leptin delivery can increase cardiac fibrosis in mice via mTOR signaling [31]. Leptin also mediates fibrosis in a variety of other organs and thus may be an important contributor to this response [32–34].

In a prior study of myeloid-specific catalase over-expression during hindlimb ischemia, impaired healing and angiogenesis was seen [21]. This was attributed to defective macrophage recruitment and inflammatory response, as well as disordered angiogenesis. In the current study we saw improved recruitment of endothelial cells and new vessel formation. While the infiltration of inflammatory cells has not been directly compared between myocardial infarction and hindlimb ischemia, it is possible that the level of cells was much more robust following MI as compared to hindlimb ischemia. Indeed, our cytokine array showed a large increase in G-CSF which may play a role in recruitment of cells. Moreover, we saw no difference in CD45-positive cell recruitment between WT and Tg^(MLC−CAT)^ mice. Additionally, as the heart is much more vascular than muscle, it is possible that increases in local collateral flow as seen in mice could allow for the migration of myeloid-lineage cells to the infarcted region. Investigating potential mechanisms, our cytokine array determined increases in VEGF, FGF, and IGF-1, all of which have been associated with increased angiogenesis and may have contributed to this response. Finally, permanent ligation was chosen as a model as it was similar to the prior study in mouse hindlimbs, as well as a clinically-relevant model. We also performed ischemia-reperfusion studies (30 min of ischemia followed by reperfusion) and saw similar trends in acute protection that was lost long-term with increases in collagen (data not shown). This is quite interesting as it suggests similar contributions or mechanisms of myeloid-derived catalase despite potentially different mechanisms of injury.

## 3. Experimental Section

### 3.1. Animals

Transgenic mice over-expressing catalase in myeloid cells (Tg^(MLC−CAT)^) were created on a C57BL/6J background as described previously [21]. Briefly, human catalase under the control of a myeloid-specific promoter Lysz was exposed by Cre/LoxP mediated excision of a green fluorescent protein and stop codon, allowing for over-expression of catalase only in cells of myeloid lineage. In Tg^(MLC−CAT)^ mice, GFP remains in all non-macrophages and is an internal control for recombination. Control mice used for all experiments, designated as wild-type (WT) mice, were negative littermates. All mice used were males between 8 and 12 weeks.

### 3.2. MI Model

Permanent ligation of the left anterior descending (LAD) artery was performed under isofluorane anesthesia to create a mouse model of MI in all mice as described previously [20]. Sham animals received thoracotomy and all subsequent procedures without placement of a ligating suture. All experimental protocols were approved by Emory University Institutional Animal Care and Use Committee (protocol DAR-2001731-042415BN valid through 24 April 2015).

### 3.3. Echocardiographic Studies

Indices of left ventricular function such as EF and FS were measured via two-dimensional (2-D) echocardiography which was performed using a high-resolution imaging system with a 30-MHz imaging transducer (Vevo 770; VisualSonics, Toronto, ON, Canada) as described previously in anesthetized mice [20]. A researcher blinded to genotypes and treatments analyzed the images. Left ventricular end-diastolic dimension (LVDd) and left ventricular end-systolic dimension (LVDs) were measured. These two indices were used to calculate percent fractional shortening (FS%), which quantifies contraction of the ventricular wall (FS% = (LVDd − LVDs)/LVDd × 100%).

### 3.4. Infarct Size Determination

Infarct size was measured three days after LAD artery ligation. Evans blue (4%, 0.3 mL) was delivered to the left ventricle via the carotid artery. The ventricle was excised and sectioned into 2-mm cross-sectional segments followed by immersion in 1% triphenyl-tetrazolium chloride (TTC). Following overnight incubation in 4% paraformaldehyde, the sections were imaged using a Nikon camera. Infarct size infarct area/area at risk) was then calculated using Image J software (National Institutes of Health, Bethesda, MD, USA).

### 3.5. Measurement of H_2_O_2_

Hydrogen peroxide production in heart tissue was measured as described previously using Amplex red assay (Invitrogen, Grand Island, NY, USA) on left ventricular tissue pieces and normalized to wet tissue weight [20].

### 3.6. Catalase Activity Assay

Catalase activity was assessed in homogenized heart tissue via decomposition of H_2_O_2_ at 240 nm utilizing a plate reader in the presence and absence of 3-amino-1,2,4-triazole (3-AT, a catalase inhibitor) and normalized to protein levels as determined by Bradford assay as previously described [35]. Other peroxidase activity was determined by the 3-AT insensitive fraction.

### 3.7. CD45 Cell Sorting

Single-cell suspensions were prepared from pooled LV tissues (*n* = 3) of both WT and Tg^(MLC−CAT)^ mice on day 7 following MI. Infarcted hearts were harvested, LV tissue isolated and minced with fine scissors and placed into a cocktail of collagenase I, collagenase XI, DNase I, and hyaluronidase (Sigma-Aldrich, St. Louis, MO, USA) while shaking at 37 °C for 1 h. Cells were then triturated through nylon mesh and centrifuged (15 min, 1500 rpm, 4 °C). Cells were washed once and re-suspended in Hank’s Balanced Salt Solution (with Ca^2+^/Mg^2+^) buffer, blocked with 1% Fetal Calf Serum, and incubated in PE-conjugated anti-mouse CD45 (Invitrogen). Cells were then sorted based on CD45 expression to identify cells of the myeloid lineage, and catalase activity assay performed on protein isolated from both the CD45^+^ and CD45^−^ cell populations.

### 3.8. Collagen Staining

Collagen deposition was determined by Picrosirius red (Sigma-Aldrich) staining, as previously described [20]. Briefly, 5-μm tissue sections were stained with Picrosirius red and imaged using light microscopy. A researcher blinded to genotypes and treatment groups measured total collagen area (areas stained red) and normalized to total LV area, using Matlab™ software (Mathworks, Natick, MA, USA).

### 3.9. Angiogenesis Staining

Endothelial cell and vessel formation were measured by Isolectin staining as previously described [17]. Briefly, 5-μm tissue sections were stained with Isolectin conjugated to Alexafluor-647 (Invitrogen) and imaged using fluorescent microscopy. Positively stained cells were counted in the infarcted tissue area and reported as either number of vessels or endothelial cells per mm^2^.

### 3.10. Cytokine Array

Protein homogenates from mice subjected to MI for 7 days were collected and 100 μg were used in a cytokine ELISA array (Signosis, Santa Clara, CA, USA) per manufacturer’s protocol. Pooled protein from 5 animals per group was used to reduce variability and data were expressed as OD450/μg protein.

## 4. Conclusions

In the current study, over-expression of catalase in myeloid-lineage cells leads to acute protection as evidenced by improvement in ventricular function, diminished levels of H_2_O_2_, increased catalase activity, increased angiogenesis and diminished infarct size. The lack of further ventricular improvement after 7 days could be attributed to the increase in fibrosis and diminished catalase activity observed at 21 days, though we found compensatory increases in other peroxidases. These data taken together with our previous findings in catalase-specific over-expression, underscore the need for consideration of spatial and temporal signaling in the context of catalase therapy.

## Figures and Tables

**Table 1.** Levels of cytokines determined by array. Values are OD450/μg protein.

Analyte	WT SHAM	WT MI	Tg^(MLC−CAT)^ SHAM	Tg^(MLC−CAT)^ MI
TNFα	1.8	3.1	2.4	2.2
IGF	1.4	2.4	1.9	3.5
VEGF	0.3	2.8	2.1	3.9
IL-6	1.3	2.1	1.9	1.9
FGF	1.5	2.1	2.4	2.4
IFN	0.9	1.4	0.8	2.1
Leptin	0.2	0.3	2.0	2.8
IL-1α	3.5	2.1	2.7	1.1
IL-1β	3.1	6.7	4.5	3.6
G-CSF	2.1	0.6	0.1	0.5
GM-CSF	2.7	0.0	2.2	0.0
MCP-1	0.0	0.8	0.0	0.4
MIP-1a	1.0	0.9	0.0	1.2
SCF	0.8	1.6	1.9	6.2
Rantes	0.0	1.6	0.8	0.4
PDGF	0.0	2.2	0.0	0.4
IL-17	2.0	2.2	0.8	1.9
IL-2	0.0	0.6	0.0	0.1
IL-4	0.0	2.4	0.2	0.8
IL-10	2.1	5.5	3.6	4.1
